# Married women pre-marital HIV testing status in Ethiopia: Individual and community level factor analysis

**DOI:** 10.3389/fmed.2023.913040

**Published:** 2023-03-02

**Authors:** Molla Yigzaw Birhanu, Daniel Bekele Ketema, Melaku Desta, Samuel Derbie Habtegiorgis, Belayneh Mengist, Alehegn Aderaw Alamneh, Ayenew Negesse Abeje, Eniyew Tegegne, Aytenew Geremew Mengist, Migbar Dessalegn, Getamesay Molla Bekele, Selamawit Shita Jemberie

**Affiliations:** ^1^Department of Public Health, College of Health Sciences, Debre Markos University, Debre Markos, Ethiopia; ^2^Department of Human Nutrition, College of Health Sciences, Debre Markos University, Debre Markos, Ethiopia; ^3^Department of Human Nutrition, College of Health Sciences, Debre Markos University, Debre Markos, Ethiopia; ^4^Department of Environmental Health, College of Health Sciences, Debre Markos University, Debre Markos, Ethiopia; ^5^Department of Gynecology and Obstetrics, College of Medicine and Health Sciences, Injibara University, Injibara, Ethiopia; ^6^Department of Surgery, School of Medicine, Debre Markos University, Debre Markos, Ethiopia; ^7^Department of Gynecology and Obstetrics, School of Medicine, Debre Markos University, Debre Markos, Ethiopia

**Keywords:** pre-marital, married women, HIV testing, Ethiopia, multi-level

## Abstract

**Introduction:**

Marriage between serodiscordant individuals accounts for 65–85% of new infections. Pre-marital Human Immune Virus (HIV) testing opens the door for HIV infection prevention and control. There are no studies that have evaluated the coverage and factors influencing pre-marital HIV testing at the community level in Ethiopia.

**Methods:**

This study was conducted using 10,008 samples of data extracted from Ethiopian demographic and health surveys (EDHS), 2016. To identify individual and community level factors a multi-level binary logistic regression model was used. Among fitted models, “full” model was taken as the best model. To declare the presence or absence of significant association with pre-marital HIV testing, a *p*-value < 0.05 with confidence interval (CI) was used.

**Results:**

In Ethiopia, 21.4% (95% CI: 20.6, 22.2%) of study participants had pre-marital HIV testing. Age 35–49 years (AOR = 0.25; 95% CI: 0.09, 0.66), educated (AOR = 1.76; 95% CI: 1.17, 2.79), rich (AOR = 1.95; 95% CI: 1.13, 3.55), having media exposure (AOR = 1.54; 95% CI: 1.30, 4.71), and high community level literacy (AOR = 0.38; 95% CI: 0.22, 0.66) were factors significantly associated with pre-marital HIV testing.

**Conclusion:**

The low coverage of pre-marital HIV testing in Ethiopia is insufficient to have a significant influence on the HIV/Acquired Immune Deficiency Syndrome (AIDS) epidemic. Information dissemination to create awareness about human rights and public health implications of pre-marital HIV testing áre necessary while it is made mandatory.

## Introduction

### Background

The Human Immune Virus/Acquired Immune Deficiency Syndrome (HIV/AIDS) is a fatal viral disease that has spread worldwide (1, 2). Women and girls account for approximately 750,000 of the 1.5 million new infections, and HIV affects 20 million women and girls out a total population of 36.0 million adults. Aside from the aforementioned, 4,200 adolescent girls and young women between the aged of 15 and 24 years are infected with HIV each week worldwide (3). However, approximately 16% (6 million people) still require HIV testing services (4). In Africa, HIV screening rates range from 33.5 to 82.3% (5–8). Sub-Saharan Africa, including Ethiopia, is home to more than three-quarters of HIV/AIDS patients, and one women are disproportionately affected (9).

Human Immune Virus testing is a public health program that focuses on screening to reduce the spread of HIV/AIDS in order to combat its impact on community and national economic output (10, 11), because HIV test is regarded as a critical entry point for HIV detection, care and treatment, prevention, and support services (6, 7). It protects people who have been infected by an HIV seropositive partner as well as their infants from HIV infection (9). Goal 3 of the Sustainable Development Goals (SDGs) focuses on “Good health and wellbeing,” with one of the key priorities being to end the HIV/AIDS epidemic by 2030. This study adds to the growing consensus that HIV/AIDS prevention and control remain critical agenda items (12). According to the prior studies, HIV testing is the most cost-effective measure for the prevention and control of HIV transmission in Africa (13, 14). The Ethiopian government has embraced pre-marital voluntary HIV counseling and testing as a key component of the country’s HIV/AIDS prevention and control efforts, providing prospective couples with the opportunity to know their HIV status before marriage (15).

A clinical trial investigation found that HIV sero-discordant couples account for around 65–85% of new infections acquired *via* a married/cohabiting partner (16). HIV positive people in sero-discordant marriages endanger HIV negative spouses (17). In the same spirit, a study conducted in Zambia and Rwanda discovered that sero-discordant couples are responsible for an estimated 50% of new heterosexual HIV infections (18). Urban residency (19), secondary and post-secondary education (20), women aged 25–34 years and 35 years and older (21), being rich in wealth index, divorced/widowed in marital status (22), drinking alcohol (23), and being unemployed (24) were the identified factors having a significant association with pre-marital HIV testing at individual level.

Knowing the pre-marital HIV testing status is critical not only for the HIV-uninfected individual, but also for the HIV-infected individual, in order to start antiretroviral prophylaxis before the immune system deteriorates and to exercise the right to marry and find a family. As a result, this multi-level analysis of determinants of pre-marital HIV testing status and associated factors among married women in Ethiopia was conducted.

## Materials and methods

### Study setting, period, and design

This study was conducted in Ethiopia using secondary data, extracted from the Ethiopian demographic and health survey (EDHS) 2016. Ethiopia is organized as a Federal Democratic Republic having nine regional states and two city administrations. It has a total of 1,100,000 km^2^, and its regional states are divided into zones, which are further subdivided into districts, which are further subdivided into kebeles, the lowest administrative divisions (25). Ethiopia is the second most populous country in Africa, after Nigeria, with a population of about 112 million people (56,010, 000 females and 56, 069, 000 males in 2019) (26). Ethiopian culture is diverse and generally organized along ethnolinguistic lines. There are over 80 ethnic groups in the country that speak different local languages (such as Amharic, Oromo, Tigrinya, and others), and English is the most commonly spoken foreign language and is taught in secondary schools and universities. According to the 2007 population and housing census (PHC), Ethiopia had 84,915 enumeration areas/clusters, 67,730 of which were rural clusters and 17,185 of which were urban clusters, with a total of 15,411, 559 households enumerated. An Enumeration Area (EA) is a geographical area with an average of 181 households. These EAs were served as a sampling frame for the 2016 EDHS survey, which was conducted in across nine regions and two administrative councils of Ethiopia from October 18, 2016 to June 27, 2016 (27). This study was conducted from January 18, 2016 to June 27, 2016.

### Eligibility criteria

All married women who were registered in the EDHS, 2016 were included in this study and married women having incomplete registration were excluded.

### Data sources and sampling procedures

The data for this analysis were derived from EDHS 2016 and obtained from the measure DHS website at http://www.dhsprogram.com. With measure DHS’s permission, the data sets were downloaded in Stata format. The study participants were drawn using a stratified, two-stage cluster design, with EAs as primary sampling units and households as secondary sampling units. Each region was divided into urban and rural clusters, with a total of 21 strata. At each of the lower administrative levels, the proportional allocation was achieved by sorting the sampling frame within each sampling stratum before sample selection, according to administrative units at different levels, and by using a probability proportional to size selection at the first stage of sampling. In the first step, 645 EA (202 EAs in urban areas and 443 EAs in rural regions) were chosen with a probability proportionate to EA size from a total list of 84,915 EAs established for the 2007 PHC (28).

### Population

The source population contained of all married reproductive age women living in Ethiopia, the study population consisted of married reproductive age women found in the selected cluster, and the study units comprised of married reproductive age women in the reproductive age group found in the selected household.

### Study variables

#### Dependent

Pre-marital HIV testing (yes/no) status of married women in the reproductive age group was the outcome/dependent variable of the study.

#### Individual-level variables

Women’s ages, Educational achievement of mothers, wealth in the family, women’s occupational status, residence, media exposure.

#### Community-level variables

We relied primarily on EA to convey aggregate-level statistics at the community level because the DHS did not collect them. As a result of aggregating individual level variables at the community (cluster) level, aggregate community-level variables were formed, and the aggregate variables were classed as low or high based on the distribution of the proportion values obtained for each cluster. Hence, media exposure, literacy, poverty, behavior, and residence were the community-level variables.

### Computation and measures of variables

#### Media use

If a respondent used any newspaper/magazine, radio, television, or internet, regardless of frequency levels, “nearly every day,” “at least once a week,” and “less than once a week” were recoded as “Yes,” while the response level “not at all” was recoded as “No.”

#### Community-level media use

Was classified as high if the proportion was 50–100%, and low if the proportion of women using media in the community was less than 50%.

#### Community-level literacy

Community-level literacy was classified as high if the proportion of women with primary, secondary, and higher education was 50–100 percent, and low if the proportion was less than 50 percent.

#### Community-level poverty

The proportion of women from the two lowest wealth quintiles in a specific community was classified as high if it was 50–100 percent, and low if it was less than 50 percent.

### Patient and public involvement

The study did not include any patients. Through the publication of study reports and open access journal articles, study findings are made available to participants and the general public. The study webpages included contact information for the research team in case anyone wanted to directly request publications.

### Data management and analysis

A multivariable multi-level analysis model was used to determine the fixed and random effects of covariates related with pre-marital HIV testing among married women in Ethiopia. We came up with four distinct models. The first model, which was an empty or unconditional model with no explanatory variables, was used to deconstruct the variance in the enumeration region (cluster). It was also employed in the fitting of a multi-level statistical application. The second model included variables at the individual level, while the third model included variables at the community level. Finally, the fourth model (Full model) took variables at both the individual and community levels into account. The statistical significance level was chosen at *p* < 0.05. The Akaike Information Criterions (AIC) were used to choose the best model from among the four options. As a result, the lower value of the Akaike Information Criterion suggests a better model fit, which was the full model. The adjusted odds ratio (AOR) was used to report the results of fixed effects (measures of association), along with 95% confidence intervals (CI) and *p*-value < 0.05. STATA version 17.0 software was used for data management and analysis.

### Ethical consideration

We used population-based secondary records from the public domain/online in this investigation. Measure DHS has given the authors permission to use the data. The DHS program adheres to criteria that protect respondents’ privacy. Data accessed from measure DHS database at http://dhsprogram.com/data/availabledatasets.cfm. We obeyed the terms and conditions of data sharing policy; data kept confidential, used for the current study only.

## Results

### Socio-demographic characteristics

As a weighted sample, 10,008 married reproductive-aged women were included in this study. About 3,007 (30.05%) of the study participants in the study were between the ages of 25 and 29. In terms of income, approximately 3,740 (37.73%) of the study participants were the poorest, and 4,927 (49.24%) were uneducated. Furthermore, 60,609 (60.55%) of them were not working in their occupation. Approximately 7,625 (76.19%) of survey participants had no media exposure (Table 1).

**TABLE 1:** Socio-demographic characteristics of the study participants.

Variables	Categories	Weighted frequency	Percentage
Age	15–19	347	3.47
	20–24	2,036	20.34
	25–29	3,007	30.05
	30–34	2,234	22.32
	35–39	1,591	15.90
	40–44	605	6.05
	45–49	188	1.88
Household wealth quintile	Poorest	3,776	37.73
	Poorer	1,918	19.16
	Middle	1,587	15.86
	Richer	1,401	14.00
	Richest	1,326	13.25
Husband’s/partner level of education	No education	4,928	49.24
	Primary	3,220	32.17
	Secondary	1,015	10.14
	Higher	845	8.44
Maternal level of education	No education	6,490	64.85
	Primary	2,486	24.84
	Secondary	671	6.70
	Higher	361	3.61
Occupation status of women	Not working	6,060	60.55
	Working	3,948	39.45
Media exposure	Yes	2,383	23.81
	No	7,625	76.19

### From 2011 to 2016, community-level characteristics of married reproductive-age women in Ethiopia

The study included 645 clusters, 269 (41.74%) of which had significant community poverty and 504 (78.19%) of which had no media exposure. Furthermore, more than 436 (67.60%) of them had a high level of community literacy, and 442 (68.54%) of them lived in rural areas (Table 2).

**TABLE 2:** Community level characteristics of the study participants.

Variables	Categories	Weighted frequency	Percentage
Poverty	Low	374	58.26
	High	268	41.74
Media exposure	Yes	140	21.81
	No	502	78.19
Literacy	Low	208	32.40
	High	434	67.60
Residency	Rural	202	31.46
	Urban	440	68.54

### Pre-marital HIV tested

**FIGURE 1:**
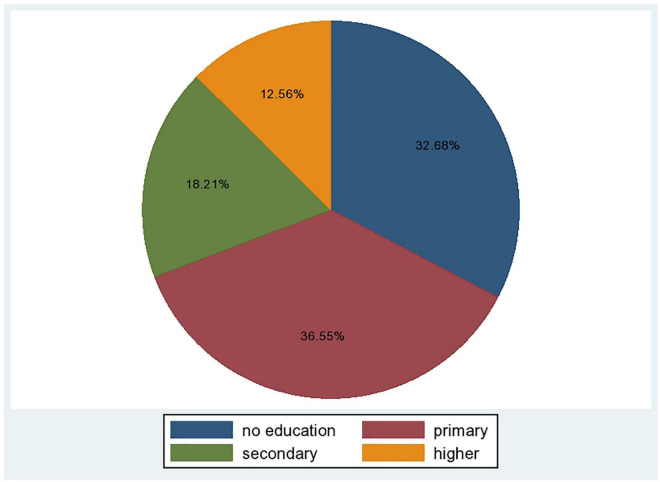
Pre-martial HIV testing over the educational status of married reproductive aged women in Ethiopia.

Pre-marital HIV testing was performed on 2,142 (21.40%) of the study participants. When we examined at the experience of pre-marital HIV testing based on women’s educational status, we found that 783 (36.55) and 700 (32.68%) of the women who underwent pre-marital HIV testing had primary education and 32.68 percent had no education, respectively (Figure 1). In terms of community media exposure, around 55% of pre-marital HIV tests had community level media exposure (Table 3).

**TABLE 3:** Pre-marital HIV testing among married women.

Variables		Pre-marital HIV testing status		Total
		**No**	**Yes**	
Age	15–19	252	95	347
	20–24	1,473	563	2,036
	25–29	2,226	781	3,007
	30–34	1,789	445	2,234
	35–49	2,126	258	2,384
Community level literacy	Low	84	124	208
	High	359	75	434
Community wealth index	Low	212	162	374
	High	231	37	268
Educational status	Not educated	5,790	700	6,490
	Educated	2,076	1,442	3,518

### Model selection and comparisons

There was high heterogeneity between clusters for pre-marital HIV testing, accounting for 57.89% of the overall variation. Pre-marital HIV testing varies due to married women living in different clusters. As a result, we decides to model the data using a nested structure. To account for the presence of nested data structures, a multi-level binary logistic regression model was considered. Table 4 displays the outcomes of the four models developed and compared using AIC.

**TABLE 4:** Model comparison and selection.

Comparison criteria	Model I	Model II	Model III	Model IV
AIC	8257.524	641.4457	7401.721	518.5607

Based on the data in Table 4, model 4, is the final model that includes both individual and community level variables with random effects, is most suited for interpretation. The statistically substantial difference in model fit (*p* < 0.001) and lower values in Model 4 provide additional statistical evidence for the suitability of the multi-level binary logistic regression model. According to the full model shown below the table, community-level variables (high community literacy, presence of community media exposure), and individual-level variables (being between the ages of 35 and 49, being educated, and being rich in wealth index) were significantly associated with pre-marital HIV testing at *P*-value < 0.05 as cutoff of point.

### Factors associated with pre-marital HIV testing

The chance of experiencing pre-marital HIV testing was 1.76 (AOR = 1.76; 95 percent CI: 1.17, 2.79) times more likely in educated married reproductive age women than in uneducated married reproductive aged women. Rich women are 1.95 (AOR = 1.95; 95 percent CI: 1.13, 3.55) times more likely than low-income women to get tested for HIV before marriage. women who have been exposed to the media are 1.54 (AOR = 1.54; 95% CI: 1.30, 4.71) times more likely to have HIV test prior to marriage. Furthermore, communities with high level of literacy are 0.62 (AOR = 0.38; 95 percent CI: 0.22, 0.66) times less likely to have HIV tested before marriage (Table 5).

**TABLE 5:** Factors associated with pre-marital women HIV testing status in Ethiopia.

Variable	Categories	AOR (95% CI)			
		**Model I**	**Model II**	**Model III**	**Model IV**
Age	15–19		1.00		1.00
	20–24		1.33 (0.96, 1.82)		1.45 (0.86, 1.84)
	25–29		0.9 (0.67, 1.35)		0.70 (0.26, 1.85)
	30–34		0.78 (0.78, 1.01)		0.60 (0.23, 0.55)
	35–49		0.36 (0.26, 0.51)*		0.25 (0.09, 0.66)*
Occupation	Not working		1.00		1.00
	Working		1.14 (0.98, 1.34		1.20 (0.80, 1.80)
Chat chewing	No		1.00		1.00
	Yes		1.55 (1.21, 1.99)*		1.82 (0.94, 3.24)
Smoking	No		1.00		1.00
	Yes		0.82 (0.35, 1.90)		2.54 (2.12, 3.98)
Wealth index	Poor		1.00		1.00
	Rich		0.98 (0.34, 1.34)		1.95 (1.13, 3.55)*
Residence	Urban		1.00	1.00	1.00
	Rural		0.17 (0.13, 0.23)*	3.54 (2.91, 4.00)*	0.69 (0.40, 1.21)
Community media exposure	No		1.0	1.00	1.00
	Yes		1.74 (1.49, 2.02)*	0.69 (5.03, 9.51)*	1.54 (1.30, 2.58)*
Community-poverty	Low		1.00	1.00	1.00
	High		1.93 (1.60, 2.31)*	0.39 (2.85, 5.22)*	0.76 (0.42, 1.40)
Community-literacy	Low		1.00	1.00	1.00
	High		2.65 (2.26, 3.11)*	3.4 (1.29, 9.50)*	0.38 (0.22, 0.66)*
Community educational status	Not educated	ICC = 0.5789		1.00	1.00
	Educated			2.39 (2.15, 4.23)	1.76 (1.17, 2.79)*

AOR-adjusted odds ratio. *Significant at *p* < 0.05, ICC = infraclass correlation.

## Discussion

This study looked at both individual and community-level factors associated with pre-marital HIV testing among married reproductive-aged women in Ethiopia who had married within the previous 5 years of the survey. About 21.4% (95% CI: 20.6, 22.2%) of women married within the 5 years of the survey in Ethiopia had had pre-marital HIV testing. This figure was lower than that found in Yining, china where 33.74% of them underwent pre-marital HIV testing (29). This is owing to the fact that in China, women have more media exposure than in Ethiopia, this high media exposure and less literacy rate in China encouraged people to get pre-marital HIV testing more than Ethiopian. That is why pre-marital HIV testing practice in Ethiopians was lower than in China.

The odds of experiencing pre-marital HIV testing among study participants aged 35–49 years was reduced by 75% (AOR: 0.25, 95% CI: 0.09, 0.66) compared to participants aged 15–19 years. It is consistent with the findings of an Iranian study on the acceptability of pre-marital HIV testing (30, 31). This could be due to the fact that as women get older, they become less influenced by others and less sexually exposed than women between the ages of 15 and 19. As a result, they were less eager to undergo pre-marital HIV testing. Hence, health professionals should promote premarital HIV testing equally among reproductive age women in order to include all reproductive age groups, particularly those aged 35 to 49.

The chance of experiencing pre-marital HIV testing among educated married women was 1.79 (AOR: 1.79, 95% CI: 1.17, 2.79) times more likely to have pre-marital HIV testing than those who were not educated. Prior research backs up this finding (32–34). This could be attributed to educated women having greater access to knowledge about pre-marital HIV testing, which allows them to know as it prevents HIV transmission from a positive to a negative spouse and mother to children, ultimately leading them to have a more positive attitude and experience pre-marital HIV testing. As a result, the minister of health should take the women’s educational backgrounds into account when promoting perimarital HIV testing among reproductive-age women in order to create awareness equity. Policymakers and programmers should prioritize uneducated women over educated women when it comes to creating work awareness about the importance of perimarital HIV testing.

The odds of pre-marital HIV testing was 1.95 (AOR: 1.95, 95 percent CI: 1.13, 3.55) times more likely among rich women as compared with poor women. This is supported by a study conducted in Cameroon which was entitled with “effect of sociodemographic and health seeking behaviors” (35–37). This could be attributed to rich women being less concerned about the cost of testing far from their home in order to avoid the stigma that is understood when one of the two is positive. As a result, wealthy women were more willing to have pre-marital HIV testing. As the government, in collaboration with non-governmental organizations, has made HIV testing payment free, it should cover the transportation costs for those who travel from afar for perimarital HIV testing to alleviate transportation issues. In collaboration with religious leaders, healthcare professionals should promote the importance of perimarital HIV testing and the associated expenses covered by the government and other stakeholders for the community at large through meeting and social media.

The odds of pre-marital HIV testing was 1.54 (AOR: 1.54, 95 percent CI: 2.30, 4.71) times more likely among women who were lived in a community with high media exposure as compared with women who were lived in a community with having low media exposure. This finding is consistent with the previous studies (38–40). This is because these women have a better chance of accessing information, which leads to pre-marital HIV testing by increasing awareness and, ultimately, acceptability of pre-marital HIV testing because media is one source of information. The government should make a concerted effort to increase the availability and accessibility of media exposure (such as local FM radio, newspapers, and television) to communities with limited media exposure, as this is very important for information dissemination to create awareness regarding perimarital HIV testing among reproductive age women.

The odds of pre-marital HIV testing for women lived in communities having high literacy was lowered by 62% (AOR: 0.38, 95% CI: 0.22, 0.66) as compared to those women lived in low literacy. This is supported by the study conducted entitled “Low Health Literacy Is associated with HIV test Acceptance” (41–43). This is because women who lived in high health literacy communities appear to be less willing to undergo pre-marital HIV testing due to their limited ability to absorb advice and information provided by healthcare workers to undertake pre-marital HIV testing when compared to women who lived in low health literacy communities. In addition to the foregoing, the minister of health incorporation of minister of education should improve the availability and accessibility of education for those communities having high literacy rates.

### Strength and limitation of the study

This study has provided a wealth of information, particularly on community-level factors influencing premarital HIV testing, which are critical for the prevention and control of HIV transmission between couples and from mother to fetus. This study relied on secondary data, which may have underestimated or overestimated the study participants’ pre-marital HIV testing status. Those scientific societies that will have the opportunity to read this research should consider this point.

## Conclusion and recommendations

The low coverage of pre-marital HIV testing among married reproductive age women in Ethiopia is insufficient to have any significant impact on the HIV/AIDS epidemic. Being between the ages of 35 and 49, and high levels of community literacy were characteristics that hampered pre-marital HIV testing, while individual factors including higher levels of education, wealth index and media exposure were factors that increased the likelihood of accepting pre-marital HIV testing. The use of different media to educate and raise awareness among all reproductive age groups may be encouraged. Governments and HIV program implementers may consider establishing guidelines and regulations for mandatory pre-marital HIV testing among all reproductive age groups, even while they recognize the human rights and privacy implications of mandatory pre-marital HIV testing and the fact that HIV can be acquired and transmitted to women post-marital as well.

## Data availability statement

The datasets presented in this study can be found in online repositories. The names of the repository/repositories and accession number(s) can be found in the article/supplementary material.

## Ethics statement

The studies involving human participants were reviewed and approved by the Major DHS. The patients/participants provided their written informed consent to participate in this study.

## Author contributions

MB: conceptualization. MB, GB, AM, DK, and ANA: data curation. MB, DK, GB, AM, ANA, and SJ: formal analysis and methodology. MB, GB, AM, DK, and ANA: software. MB, GB, AM, SH, ANA, and SJ: supervision. MB, GB, AM, and SJ: validation. MB, DK, and SJ: visualization. MB, SJ, DK, GB, AM, and SH: writing original draft. MB, GB, AM, DK, SH, ANA, and SJ: writing review and editing. All authors contributed to the article and approved the submitted version.
